# Temporal Trends of Bleeding Episodes during Half- vs. Standard-Dose Ticagrelor in Acute Coronary Syndrome Patients with Low Platelet Reactivity: A Randomized BLEEDING-ACS Trial

**DOI:** 10.3390/jcm10061159

**Published:** 2021-03-10

**Authors:** Laeun Kim, Jeong Cheon Choe, Jin Hee Ahn, Hye Won Lee, Jun-Hyok Oh, Jung Hyun Choi, Han Cheol Lee, Kwang Soo Cha, Taek Jong Hong, Young-Hoon Jeong, Jin Sup Park

**Affiliations:** 1 Department of Cardiology and Medical Research Institute, Pusan National University Hospital, Busan 49241, Korea; laeun0315@gmail.com (L.K.); purefountain@naver.com (J.C.C.); reinee81@naver.com (J.H.A.); lhw1400@hanmail.net (H.W.L.); jhoh724@hanmail.net (J.-H.O.); mariahyeon@gmail.com (J.H.C.); glaraone@hanmail.net (H.C.L.); chakws1@hanmail.net (K.S.C.); bestdoctorprofessor@gmail.com (T.J.H.); 2 Department of Internal Medicine, Gyeongsang National University School of Medicine, Jinju 52727, Korea; goodoctor@naver.com; 3 Cardiovascular Center, Gyeongsang National University Changwon Hospital, Changwon 51472, Korea

**Keywords:** acute coronary syndrome, bleeding, half-dose ticagrelor, platelet reactivity

## Abstract

To assess the temporal trends of bleeding episodes during half- vs. standard-dose ticagrelor in acute coronary syndrome (ACS) patients with low platelet reactivity (LPR) during standard-dose ticagrelor (90 mg bid). ACS Patients with LPR (<85 P2Y_12_ reaction units) (*n* = 122) were randomly assigned to receive either half-dose (45 mg bid) or standard-dose ticagrelor (90 mg bid). The primary endpoint was incidence of Bleeding Academic Research Consortium (BARC) bleeding at 1 week, 1, 3 and 6 months. Dyspnea and ischemic events were also evaluated. Bleeding episodes were most commonly observed at 1 month and then decreased over time. Half-dose ticagrelor did not reduce any BARC bleeding (odds ratio [OR] 0.900, 95% confidence interval [CI] 0.563–1.440, *p* = 0.661). However, serious bleeding (BARC type ≥2) occurred less often in half-dose ticagrelor (OR 0.284, 95% CI 0.088–0.921, *p* = 0.036). The rate of moderate-to-severe dyspnea was highest at 1 month, then decreased over time. Half-dose ticagrelor did not decrease moderate-to-severe dyspnea (Borg scale ≥ 3) (OR 1.066, 95% CI 0.322–3.530, *p* = 0.916). The risk of ischemic events was also similar between the groups. In conclusions, compared with standard-dose ticagrelor, half-dose ticagrelor reduced serious bleeding events during early period of dual-antiplatelet therapy in ACS patients with LPR; however, the risk of any bleeding events and dyspnea did not differ according to ticagrelor dose. Clinical registration: KCT0004640.

## 1. Introduction

Dual antiplatelet therapy (DAPT) is an essential treatment in acute coronary syndrome (ACS) patients undergoing percutaneous coronary intervention (PCI) for at least 12 months. Current guidelines [1,2] recommend the use of a potent P2Y_12_ receptor inhibitor as a standard treatment for ACS patients [3,4]. These potent inhibitors are associated with a reduction in ischemic events and an increase in bleeding events. Therefore, a de-escalation strategy for reducing major bleeding has been studied [5]. In particular, East Asian population exhibits the so-called “East Asian Paradox” [6], where East Asian patients are more vulnerable to bleeding events and relatively resistant to ischemic events during antithrombotic treatment compared to Caucasian patients. Therefore, finding an optimal balance between risk and benefit is a challenging issue.

Among de-escalation strategies, reducing the dose of potent P2Y_12_ inhibitors could be one of the options in ACS patients with high bleeding risk and/or low platelet reactivity (LPR) [7]. In Japanese patients presented with ACS, uniform reduction of prasugrel dose with 3.75 mg is associated with a low incidence of ischemic events without increase of serious bleeding compared to clopidogrel with 75 mg [8]. Compared with standard-dose prasugrel, application of half-dose prasugrel in ACS patients with LPR reduced the rate of any bleeding by 45% during 1 month [9].

However, there is a lack of evidence supporting clinical usefulness of reduced-dose ticagrelor for ACS patients. A meta-analysis for low-dose ticagrelor, compared to clopidogrel, showed comparable bleeding events [10]. In addition, ticagrelor dose-dependent pharmacodynamic profile [11] and bleeding risk [12] in patients with prior myocardial infarction (MI) may indicate that low dose of ticagrelor can be applicable in the selected patients. However, ticagrelor of 60 mg twice daily was associated with a higher rate of major bleeding in East Asian patients [13], which observation would be related with increased pharmacokinetic profile of ticagrelor and intrinsic bleeding tendency in these subjects [9]. Thus, there is still a concern about the optimal dose of ticagrelor in East Asian ACS patients.

We conducted a randomized trial to compare the temporal trends of bleeding, dyspnea and ischemic events during half- (45 mg twice a day) vs. standard-dose (90 mg twice a day) ticagrelor administration in ACS patients with on-ticagerlor LPR, which may provide an important background for clinical application of half-dose ticagrelor in East Asian patients with ACS.

## 2. Methods

The BLEEDING-ACS (BLEEDING episodes during half- vs. standard-dose ticagrelor in Acute Coronary Syndrome patients with low platelet reactivity; Clinical registration: URL: http://cris.nih.go.kr/ (accessed on 29 January 2021), unique identifier: KCT0004640) study was conducted as a single-center, prospective, open-label, randomized trial. The study focused on assessing the temporal trends of bleeding episodes during half- vs. standard-dose ticagrelor in ACS patients, who had LPR during standard-dose ticagrelor (90 mg twice a day). The study design was approved by the institutional review board of Pusan National University Hospital (Busan, Korea). The study was performed in accordance with the principles established in the Declaration of Helsinki [14], and informed consent was obtained from all enrolled patients.

### 2.1. Patients

Inclusion criteria were ACS patients who were at least 18 years of age, who underwent successful coronary revascularization (if needed) and obtained LPR before discharge. Platelet function was performed between 3 and 5 days after PCI or coronary angiography (CAG) in patients received standard-dose ticagrelor and aspirin.

### 2.2. Study Design

Suspected ACS patients received a loading dose of 300 mg aspirin and 180 mg ticagrelor before CAG, followed by a maintenance dose of 100 mg aspirin daily and 90 mg ticagrelor twice a day during hospitalization. Established ACS patients with LPR after CAG or PCI were randomly assigned, in a 1:1 ratio, to half-dose (45 mg twice a day) or standard-dose (90 mg twice a day) ticagrelor after discharge. Randomization was performed by computer-generated block randomization at the end of the day of hospital stay. PCI was performed according to the standard method. Enrolled patients received optimal medical therapy, including high-dose statin, beta-blocker, and angiotensin blockade if indicated. DAPT was maintained for at least 1 year from the randomization.

Clinical follow-up was performed at 1 week, 1 month, 3 months, and 6 months after the index hospitalization. Information about the use of aspirin and ticagrelor, concomitant medication, and clinical status was obtained at each follow-up visit. All bleeding events and the degree of dyspnea were evaluated using the dedicated questionnaires by a dedicated coordinator.

To define on-ticagrelor LPR phenotype before discharge (post-PCI 3–5 days), VerifyNow P2Y_12_ assay (Accriva, San Diego, CA, USA) [7] was used to measure platelet function, which is a point-of-care assay based on a whole blood sample. Between 2 and 6 h after the oral intake of the last-dose ticagrelor, blood sampling was obtained through the antecubital vein into 3.2% Citrate Vacuette tubes (Greiner Bio-One Vacuette North America, Inc., Monroe, NC, USA). LPR was defined as ‘PRU < 85′ based on the consensus document [7].

### 2.3. Endpoints

The primary endpoint was the temporal trends of Bleeding Academic Research Consortium (BARC) bleeding events (type 1–5) at each clinical visit (1 week, 1, 3 and 6 months). Secondary endpoints were: (1) the temporal trends of serious bleeding (≥BARC type 2); and (2) dyspnea (Borg category-ratio [CR] 10 scale [15]). Bleeding events were evaluated using the BARC bleeding-questionnaire [16], and degrees of dyspnea were checked with the Borg CR 10 scale (mild: 0–2, moderate: 3–4, and severe: ≥5).

### 2.4. Statistical Analysis

Presently, there has not been a reliable data of bleeding during half-dose ticagrelor treatment. Based on a previous report during prasugrel therapy [9], we assumed that the incidence of BARC bleeding (any type) would be 35% and 24% during standard- and half-dose ticagrelor, respectively. We calculated the sample size (*n* = 114: 57 patients per group) required to detect assumed difference on a two-sided significance level of 0.05 with a statistical power of 80%. With assuming a drop-out risk of 10% during follow-up, 126 patients were initially enrolled.

Continuous variables were compared using Student’s *t*-test or Wilcoxon rank-sum test, while categorical variables were analyzed using the chi-squared or Fisher’s exact test. Trend comparison between groups was performed using the generalized estimating equations method. The generalized estimating equations (GEE) procedure extends the generalized linear model to allow for analysis of repeated measurements or other correlated observations, such as clustered data [17]. GEE model constructed using a completely general correlation matrix (unstructured) and binary logistic or Poisson log-linear distribution as appropriate. Half-dose ticagrelor for bleeding and dyspnea events was used to build model effect as the main effect, and parameter estimation was done by a hybrid method in which Fisher scoring iterations are performed before switching to the Newton-Raphson method, and type III analysis was used. The parameters estimation was described as odds ratio (OR) and 95% confidence interval (CI). In addition, GEE models with unstructured and binary logistic distribution were used to identify independent predictors of bleeding and dyspnea. To find a correlation between bleeding and dyspnea, another GEE models were built. The hazard ratio of ischemic events between groups was derived using the Cox proportional hazard model. Two-sided *p*-values < 0.05 were considered statistically significant. SPSS version 25 was used to perform statistical analyzes.

## 3. Results

### 3.1. Study Participants

**Figure 1:**
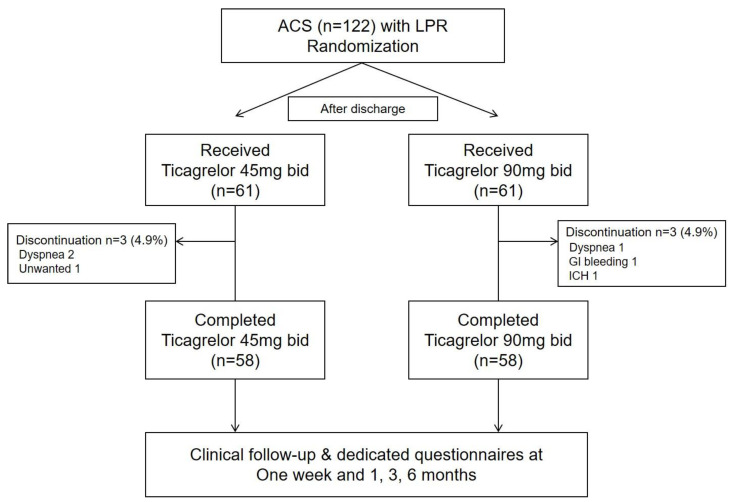
Study flow. ACS = acute coronary syndrome; GI = gastrointestinal; ICH = intracranial hemorrhage; LPR = low platelet reactivity (defined by P2Y_12_ reaction unit <85).

Between May 2017 and December 2019, a total of 122 patients were included for the final analysis, with exclusion of 4 patients due to withdrawal of informed consent. Details about study flow are shown in Figure 1. After discharge, 61 patients were randomized to receive half-dose ticagrelor, and the others to standard-dose ticagrelor. During the follow-up, three patients from each group prematurely discontinued the study drug, resulting in completion of the study protocol in a total of 116 patients (58 patients for each group).

Clinical and procedural characteristics, and discharge medications were well-balanced between groups (Table 1). About 30% of patients were diagnosed with ST-segment elevation MI, and a half had multivessel disease. Most of the patients (96.7%) were treated with PCI.

### 3.2. Bleeding Endpoints

**Figure 2:**
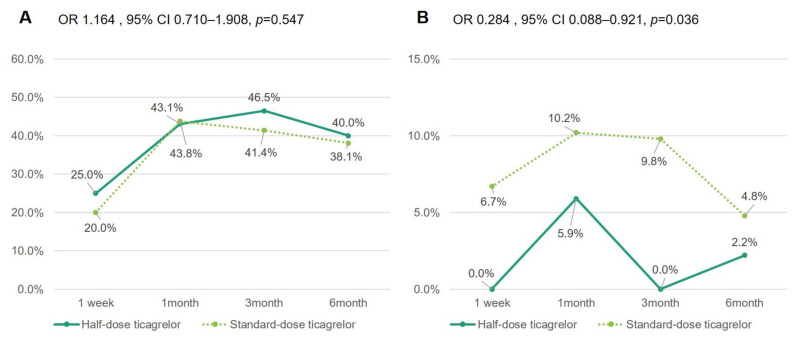
Incidence and temporal change of nuisance bleeding (BARC type 1) (**A**) and serious bleeding (BARC type ≥2) (**B**) over time during half- vs. standard-dose ticagrelor. Exponentiation of the B coefficient was calculated with standard-dose ticagrelor as a reference. BARC = Bleeding Academic Research Consortium; CI = confidence interval; OR = odds ratio.

In the entire cohort, about 25% of any BARC bleeding occurred at 1 week after discharge. The incidence of any BARC bleeding event had increased at 1 month (51.5%), then slightly decreased at 3 (48.8%) and 6 months (42.5%). The incidence of BARC type 1 bleeding between the half- and standard-dose groups did not differ at each visit (OR 1.164, 95% CI 0.710–1.908, *p* = 0.547): 25.0% vs. 20.0% at 1 week, 43.1% vs. 44.0% at 1 month, 46.5% vs. 41.5% at 3 months, and 40.0% vs. 38.1% at 6 months, respectively. However, serious bleeding (BARC type ≥2) events occurred less frequently in half- vs. standard-dose ticagrelor at every visit (OR 0.284, 95% CI 0.088–0.921, *p* = 0.036): 0% vs. 6.7% at 1 week, 5.9% vs. 10.2% at 1 month, 0% vs. 9.8% at 3 months, and 2.2% vs. 4.8% at 6 months, respectively (Table 2 and Figure 2). Two BARC type 3 bleeding events (gastrointestinal bleeding and intracranial hemorrhage) occurred within 3 months, only in the standard-dose ticagrelor. Especially, patients over 65 years old (OR 0.116, 95% CI 0.034–0.820, *p* = 0.028) and those with BMI under 25 kg/m^2^ (OR 0.097, 95% CI 0.012–0.772, *p* = 0.028) showed a lower risk of serious bleeding (BARC type ≥2) during the half- vs. standard-dose ticagrelor treatment.

To determine the independent variables related with serious bleeding (BARC type ≥2) occurrence, we performed the multivariate analysis including covariates with *p* value < 0.1 in the univariate analysis. Age (OR 1.052, 95% CI 1.012–1.094, *p* = 0.011) and half-dose ticagrelor (OR 0.305, 95% CI 0.095–0.980, *p* = 0.046) remained as independent predictors for serious bleeding events (Table 3).

### 3.3. Dyspnea and Ischemic Endpoints

**Figure 3:**
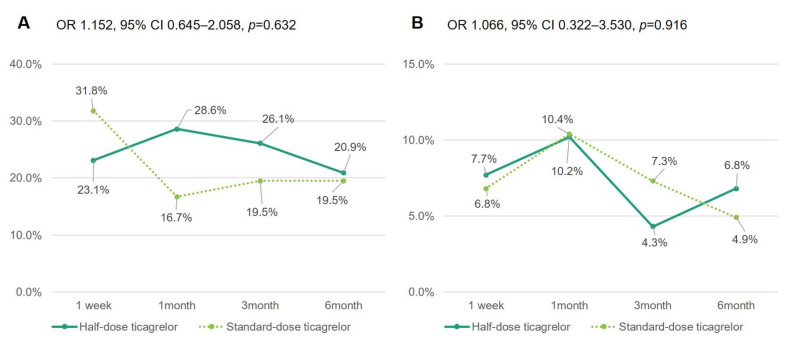
Incidence and temporal change of mild dyspnea (Borg score 1–2) (**A**) and moderate-to-severe dyspnea (Borg score ≥ 3) events (**B**) over time during half- vs. standard-dose ticagrelor. Borg score measured Borg scale category-ratio 10. Exponentiation of the B coefficient was calculated with standard-dose ticagrelor as a reference. CI = confidence interval; OR = odds ratio.

The overall incidence of dyspnea increased in the early period, from 1 week (34.4%) to 1 month (35.1%) and then decreased at 3 months (29.9%) and maintained up to 6 months (26.2%). The incidence of moderate-to-severe dyspnea (Borg scale ≥3) between the groups did not differ (OR 1.066, 95% CI 0.322–3530, *p* = 0.916): 7.7% on half-dose ticagrelor vs. 6.8% on standard-dose ticagrelor at 1 week, 10.2% vs. 10.4% at 1 month, 4.3% vs. 7.3% at 3 months, and 6.8% vs. 4.9% at 6 months, respectively (Table 2 and Figure 3). Half- vs. standard-dose ticagrelor was not associated with any dyspnea events (mild, moderate or severe) or any dyspnea scale by Borg CR 10 (Table 2). Discontinuation due to dyspnea occurred in two patients (3.3%) on half-dose ticagrelor and one patient (1.6%) on standard-dose ticagrelor. Age, hypertension and baseline creatinine level showed associations with any dyspnea events. In the multivariate analysis, only age remained as an independent predictor of dyspnea (OR 1.035, 95% CI 1.002–1.068, *p* = 0.037) (Table 3). No variables could be found for the predictor of moderate-to-sever (mod-severe dyspnea) (Supplement Table S1).

Composite of ischemic events occurred only in four patients during 6 months: two patients (3.3%) in the half- and standard-dose groups (hazard ratio 1.005, 95% CI 0.141–7.131, *p* = 0.996). Two patients received additional stent implantation due to myocardial infarction, and one patient was treated with stent implantation due to presenting unstable angina. Heart failure occurred in one patient receiving standard-dose ticagrelor (Supplement Table S2).

### 3.4. Relationship between Bleeding Event and Dyspnea

Any dyspnea event was associated with any bleeding (BARC type 1–5) (OR 1.598, 95% CI 1.008–2.533, *p* = 0.046), but not with serious bleeding (BARC type ≥2) (OR 1.378, 95% CI 0.739–2.571, *p* = 0.313). Moderate-to-severe dyspnea did not show associations with serious bleeding events (OR 0.530, 95% CI 0.010–27.945, *p* = 0.754) (Supplement Table S3).

## 4. Discussion

This is the first randomized study to determine the temporal trends of the incidence of bleeding and dyspnea events during half- vs. standard-dose ticagrelor in ACS patients with LPR. The key findings of the study are as below; (1) serious bleeding (BARC type ≥ 2) was occurred less frequently during 6 months on half- vs. standard-dose ticagrelor, especially in patients with old age and/or low BMI; (2) the rate of dyspnea did not differ according to ticagrelor dose; (3) old age was associated with both bleeding and dyspnea events. However, the relationship between bleeding and dyspnea appeared limited. A large-scale randomized trial may be needed to confirm the role of half-dose ticagrelor as a de-escalation strategy in ACS patients with LPR.

It is essential to identify the risk of bleeding during DAPT in ACS patients after PCI. P2Y_12_-directed platelet-function test (PFT) is recommended to find the therapeutic window of P2Y_12_ inhibition, and LPR phenotype may be used to find ACS patients with high bleeding risk (HBR) [18]. If ACS patients had LPR after PCI, de-escalation of P2Y_12_-inhibiting therapy (switching to moderate P2Y_12_ inhibition or reduced dose of P2Y_12_ inhibition) can be applicable to reduce bleeding events [7]. There are accumulating clinical evidence that East Asian patients experienced fewer atherothrombotic clinical events and more bleeding events during antithrombotic treatment than Caucasians [19,20]. Besides, the cut-off value of LPR in the East Asian population might be different from that in Caucasians: 126–139 PRU in East Asians vs. 85 PRU in Caucasians [21]. The higher bleeding risk in East Asian population could be explained by the increased rate of Helicobacter pylori infection (2 times higher) [22], frequent atherosclerosis of cerebral arteries, and increased hemorrhagic transformation [23] compared with Caucasian population. Therefore, the antithrombotic strategies in East Asian patients presented with ACS should be more focused on minimizing the risk of bleeding, especially in subjects with LPR.

There is a lack of large-size randomized clinical trials to evaluate clinical benefit of half-dose ticagrelor in ACS patients. In the clinical trials including stabilized MI patients [11], 60-mg ticagrelor showed a better safety profile in terms of serious bleeding (Thrombolysis in Myocardial Infarction [TIMI] bleeding: 2.3% vs. 2.6%, *p* < 0.001; TIMI minor bleeding: 1.18% vs. 1.31%, *p* < 0.001) despite no differences in post-dose PRU between the regimens [13]. However, the incidence of TIMI major bleeding was higher in Asian patients even during 60-mg ticagrelor (3.74% vs. 2.3%) [12]. Thus, there is still a concern about the optimal-dose ticagrelor in East Asian patients. Our study showed that half-dose ticagrelor had better safety profile even in ACS patients with LPR during standard-dose ticagrelor; these findings might be a cornerstone to find the optimal de-escalation strategy for reducing bleeding events in ACS patients with HBR phenotype.

In a clinical study from Japan [8], the incidence of overall and TIMI bleeding events were 49.8% and 9.6%, respectively, in ACS patients treated with adjusted-dose prasugrel (maintenance dose, 3.75 mg). The incidence of overall bleeding appeared somewhat higher in our study compared with previous Japanese report (70% vs. 49.8%). These findings could be explained by the fact that our participants had baseline low level of PRU, which had a high propensity for HBR [18]. Also, a dedicated questionnaire for bleeding was conducted at every visit. Overall, nuisance bleeding events after initiating DAPT occurred more frequently and earlier. Minor bleeding was associated with cessation of antiplatelet therapy even though major bleeding might be one of the most common cause to discontinue the antiplatelet agent [24]. Furthermore, antiplatelet discontinuation should be a critical issue to increase the risk of ischemic event after PCI [25]. Patients with LPR should be monitored with a close observation for the drug adherence and bleeding episodes, especially during the early period of DAPT including potent P2Y_12_ inhibitor.

In our analysis, half-dose ticagrelor did not reduce any dyspnea or moderate-to-severe dyspnea. Previous studies reported ticagrelor dose-dependent incidence of dyspnea (from 10% to 20%) [26,27,28]. However, a recent meta-analysis did not find the close association between risk of dyspnea and ticagrelor dose [29]. The overall incidence of any dyspnea was much higher in our study. It might be driven by higher level in terms of active metabolite of ticagrelor (up to 50%) in East Asians vs. Caucasian [30] and a detailed questionnaire conducted by a dedicated coordinator. Discontinuation of ticagrelor due to dyspnea varied from 0.9% to 21.4% [3,31]. In our study, the rate of discontinuation due to dyspnea was only 2.4% throughout the entire study period. In addition, the severity of dyspnea decreased over time, and most of the cases were well-tolerable. Therefore, dyspnea might not be an issue in ACS patients who received a various dose of ticagrelor. However, the occurrence of dyspnea events was associated with bleeding events; thus, patients who suffered from dyspnea might require medical attention for occurrence of bleeding events.

Clinical efficacy of low dose-ticagrelor seemed promising even in high-risk ACS patients [11]. However, our study was performed in limited number of patients during the short-term follow-up. Large-scale clinical trials with long-term follow-up are required to confirm clinical benefit of half-dose ticagrelor in ACS patients. Several clinical trials conducted from East Asian countries already have suggested that any type of de-escalation strategy (e.g., early discontinuation of aspirin, uniform application of reduced-dose prasugrel, and early switching with clopidogrel) can be beneficial in reducing bleeding episodes without increase of ischemic events during DAPT with potent P2Y_12_ inhibitor [32,33,34].

Clinical implications: A de-escalation antiplatelet therapy of potent P2Y_12_ inhibitor would be required for fragile ACS patients with HBR, which strategy may be mandatory in East Asian patients with unique clinical features [8,19,20]. This BLEEDING-ACS trials evaluated clinical usefulness of PFT-guided ticagrelor de-escalation in East Asian patients presented with ACS. Compared with standard-dose ticagrelor, half-dose ticagrelor could reduce the rate of serious bleeding during 6 months, but the risk of dyspnea did not differ between the regimens. Large-scale clinical trial with long-term (~1 year) follow-up must be needed to confirm this observation and change clinical guideline for East Asian patients to maximize the net clinical benefit.

Study limitations: First, patients enrolled from a single center received open-label study drug and physicians were not blinded to randomization. However, the episodes of bleeding and dyspnea were evaluated with a dedicated questionnaires supported by an attending coordinator. Second, we enrolled ACS patients with LPR. Therefore, it is difficult to generalize the main results of this study to all ACS population. However, as LPR could represent HBR phenotype, we should consider the reduced-dose ticagrelor when treated ACS patients with LPR having other HBR profiles, such as old age. Third, the primary endpoint also included nuisance bleeding (BARC type 1), but this bleeding increased the risk of early discontinuation during DAPT [24]. Fourth, follow-up duration (6 months) was relatively short to evaluate the risk of bleeding event. Nevertheless, the major bleeding events mostly occurred within 3 months, and serious bleeding episodes decreased after 1 month. Finally, LPR cutoff (<85 PRU) was defined by consensus document derived from Western data [7] since there have been no convincing LPR cutoffs for East Asians. Because follow-up measurement of PRU was not conducted, it would be impossible to evaluate the association between PRU level and clinical events.

## 5. Conclusions

Compared with standard-dose ticagrelor, half-dose ticagrelor minimized serious bleeding (BARC type ≥ 2), especially in patients with old age and/or low BMI. This study suggests the half-dose ticagrelor could be an option of de-escalation strategy in ACS patients having LPR without increased risk of ischemic events during the early period of DAPT maintenance.

## Figures and Tables

**Table 1:** Baseline characteristics.

Characteristics	All Population (*n* = 122)	Half-Dose Ticagrelor (*n* = 61)	Standard-Dose Ticagrelor (*n* = 61)	*p* Value
Age, years (SD)	61.8 (11.3)	60.7 (1.0)	62.9 (11.6)	0.282
Male, *n* (%)	99 (81.1)	48 (78.7)	51 (83.6)	0.643
Body mass index, kg/m^2^ (SD)	25.1 (3.3)	25.3 (3.4)	24.9 (3.1)	0.533
Hypertension, *n* (%)	65 (53.3)	32 (52.5)	33 (54.1)	1.000
Diabetes mellitus, *n* (%)	33 (27.0)	14 (23.0)	19 (31.1)	0.415
Dyslipidemia, *n* (%)	23 (18.9)	9 (14.8)	14 (23.0)	0.355
Prior myocardial infarction, *n* (%)	11 (9.0)	4 (6.6)	7 (11.5)	0.527
Prior revascularization, *n* (%)	14 (11.5)	6 (9.8)	8 (13.1)	0.776
Chronic kidney disease, *n* (%)	5 (4.1)	2 (3.3)	3 (4.9)	1.000
Current smoking, *n* (%)	59 (48.4)	27 (44.3)	32 (52.5)	0.469
Clinical presentations, *n* (%)				
STEMI	36 (29.5)	22 (36.1)	14 (23.0)	0.165
NSTE-ACS	86 (70.5)	39 (63.9)	47 (77.0)	
Laboratory findings				
Hemoglobin, g/dL (SD)	14.1 (2.3)	14.1 (2.4)	14.0 (2.3)	0.763
Creatinine, mg/dL (SD)	1.82 (9.34)	0.97 (0.49)	2.67 (13.19)	0.318
Low density lipoprotein, mg/dL (SD)	131.1 (72.0)	127.3 (39.2)	134.8 (93.6)	0.573
P2Y_12_ reaction unit (SD)	31 (29)	27 (30)	35 (27)	0.109
Left ventricular ejection fraction, % (SD)	53.83 (8.0)	54.6 (8.3)	53.0 (7.7)	0.302
Procedural characteristics				
Percutaneous coronary intervention, *n* (%)	118 (96.7)	60 (98.4)	58(95.1)	0.619
Trans-radial approach, *n* (%)	97 (79.5)	49 (80.3)	48 (78.7)	1.000
Intravascular ultrasound, *n* (%)	43 (35.2)	22 (36.1)	21 (34.4)	1.000
Stent max diameter, mm	3.16 (0.72)	3.21 (0.61)	3.11 (0.81)	0.451
Stent total length, mm	37.0 (25.3)	37.5 (24.0)	36.6 (26.8)	0.851
Type of polymer in DES, *n* (%)				
Durable polymer	84 (70.0)	43 (70.5)	41 (69.5)	0.944
Biodegradable polymer	31 (25.8)	15 (24.6)	16 (27.1)	
Multi-vessel disease, *n* (%)	58 (47.5)	29 (47.5)	29 (47.5)	1.000
Target lesion, *n* (%)				
Left main	10 (8.2)	7 (11.5)	3 (4.9)	0.322
Left anterior descending	74 (60.7)	41 (67.2)	33 (54.1)	0.195
Left circumflex	37 (30.3)	18 (29.5)	19 (31.1)	1.000
Right coronary	32 (26.2)	15 (24.6)	17 (27.9)	0.837
Calcified lesion, *n* (%)	20 (16.4)	11 (18.0)	9 (14.8)	0.807
Bifurcation lesion, *n* (%)	5 (4.1)	4 (6.6)	1 (1.6)	0.361
Thrombotic lesion, *n* (%)	37 (30.3)	15 (24.6)	22 (36.1)	0.237
Discharge medications				
Beta blocker, *n* (%)	95 (77.9)	46 (75.4)	49 (80.3)	0.663
Calcium channel blocker, *n* (%)	15 (12.3)	6 (9.8)	9 (14.8)	0.581
ACEi or ARB, *n* (%)	105 (86.1)	54 (88.5)	51 (83.6)	0.601
Statin, *n* (%)	121(99.2)	60(98.4)	61(100)	0.597

Values are presented as mean (SD) or *n* (%). ACEi = angiotensin-converting enzyme inhibitor; ARB = angiotensin receptor blocker; DES = drug-eluting stent; IVUS = intravascular ultrasound; NSTE-ACS = non-ST segment elevation-acute coronary syndrome; STEMI = ST-segment elevation myocardial infarction.

**Table 2:** Comparisons of primary and secondary endpoints in half vs. standard-dose ticagrelor during 6 months.

End Points	Regimen	OR (95% CI)	*p* Value
BARC 1–5 bleeding,	Half-dose ticagrelor	0.900 (0.563–1.440)	0.661
	Standard-dose ticagrelor	1	
BARC bleeding (type 1)	Half-dose ticagrelor	1.164 (0.710–1.908)	0.547
	Standard-dose ticagrelor	1	
BARC bleeding (type ≥2)	Half-dose ticagrelor	0.284 (0.088–0.921)	0.036
	Standard-dose ticagrelor	1	
Any dyspnea events *	Half-dose ticagrelor	1.112 (0.606–2.041)	0.733
	Standard-dose ticagrelor	1	
Any dyspnea scale **	Half-dose ticagrelor	1.061 (0.537–2.094)	0.866
	Standard-dose ticagrelor		
Mild dyspnea	Half-dose ticagrelor	1.152 (0.645–2.058)	0.632
	Standard-dose ticagrelor	1	
Moderate dyspnea	Half-dose ticagrelor	1.158 (0.435–3.085)	0.769
	Standard-dose ticagrelor	1	
Severe dyspnea	Half-dose ticagrelor	0.542 (0.089–3.304)	0.507
	Standard-dose ticagrelor	1	
Mod-severe dyspnea	Half-dose ticagrelor	1.066 (0.322–3.530)	0.916
	Standard-dose ticagrelor	1	

* as binary events, ** as Borg scale category-ratio 10. BARC = bleeding academic research consortium; CI = confidence interval; OR = odds ratio.

**Table 3:** Independent predictors of bleeding and dyspnea events.

BARC Bleeding (Type ≥ 2)	Univariate		Multivariate	
	OR (95% CI)	*p* Value	OR (95% CI)	*p* Value
Age	1.058 (1.013–1.104)	0.010	1.052 (1.012–1.094)	0.011
Body mass index	0.941 (0.771–1.148)	0.549		
Hypertension	1.350 (0.447–4.072)	0.594		
Diabetes	1.314 (0.406–4.254)	0.649		
Chronic kidney disease	2.149 (0.316–14.597)	0.434		
STEMI	0.388 (0.106–1.425)	0.154		
Multi-vessel disease	0.810 (0.270–2.432)	0.708		
Hemoglobin	0.926 (0.786–1.092)	0.361		
Creatinine	0.987 (0.962–1.012)	0.298		
P2Y_12_ reaction unit	1.013 (0.989–1.039)	0.283		
Half-dose ticagrelor	0.284 (0.088–0.921)	0.036	0.305 (0.095–0.980)	0.046
**Any Dyspnea Events**	**Univariate**		**Multivariate**	
	**OR (95% CI)**	***p* Value**	**OR (95% CI)**	***p* Value**
Age	1.038 (1.006–1.072)	0.020	1.035 (1.002–1.068)	0.037
Body mass index	1.068 (0.972–1.172)	0.171		
Hypertension	1.819 (0.992–3.337)	0.053	1.531 (0.830–2.823)	0.173
Diabetes	0.995 (0.483–2.051)	0.989		
Chronic kidney disease	0.639 (0.106–3.837)	0.624		
STEMI	1.105 (0.595–2.051)	0.752		
Multi-vessel disease	1.546 (0.842–2.836)	0.160		
Hemoglobin	0.939 (0.840–1.049)	0.267		
Creatinine	0.972 (0.941–1.004)	0.084	0.964 (0.906–1.027)	0.258
P2Y_12_ reaction unit	0.990 (0.979–1.002)	0.100		
Half-dose ticagrelor	1.112 (0.606–2.041)	0.733		

BARC = Bleeding Academic Research Consortium; CI = confidence interval; OR = odds ratio; STEMI = ST-segment elevation myocardial infarction.

## Supplementary Materials

The following are available online at https://www.mdpi.com/2077-0383/10/6/1159/s1, Table S1. Independent predictors for moderate to severe dyspnea (Borg scale ≥ 3), Table S2. Comparison of ischemic endpoints between groups by Cox proportional regression analysis, Table S3. Relationship between bleeding events and dyspnea.
