# Supplementary material for: Temporal Trends of Bleeding Episodes during Half- vs. Standard-Dose Ticagrelor in Acute Coronary Syndrome Patients with Low Platelet Reactivity: A Randomized BLEEDING-ACS Trial
Source: J Clin Med. 2021 Mar 10;10(6):1159. doi: 10.3390/jcm10061159 (PMC8000097; doi:10.3390/jcm10061159)
Supplement: Supplementary file 1 [file jcm-10-01159-s001.pdf]

Supplementary Materials:

Table S1. Independent predictors for moderate to severe dyspnea (Borg scale ≥ 3).

Moderate-to-severe dyspnea	Univariate	
	OR (95% CI)	*p* Value
Age	1.022 (0.969–1.077)	0.429
Body mass index	1.106 (0.983–1.245)	0.093
Hypertension	2.413 (0.843–6.911)	0.101
STEMI	0.493 (0.089–2.733)	0.419
Multi-vessel disease	2.181 (0.772–6.163)	0.141
Hemoglobin	0.915 (0.816–1.027)	0.132
Creatinine	0.820 (0.227–2.955)	0.761
$P2Y_{12}$ reaction unit	0.983 (0.960–1.007)	0.171
Half-dose ticagrelor	1.066 (0.322–3.530)	0.916

CI = confidence interval; OR = odds ratio; STEMI = ST-segment elevation myocardial infarction..

Table S2. Comparison of ischemic endpoints between groups by Cox proportional regression analysis.

Endpoints, n (%)	Half-dose ticagrelor (n=61)	Standard-dose ticagrelor (n=61)	HR (95% CI)	*p* Value
Ischemic events	2 (3.3)	2 (3.3)	1.005 (0.141–7.131)	0.996
Myocardial infarction	1 (1.6)	1 (1.6)	1.000 (0.063–15.988)	1.000
Any revascularization	2 (3.3)	1 (1.6)	2.012 (0.182–22.190)	0.568
Target lesion	1 (1.6)	1 (1.6)	1.000 (0.063–15.988)	1.000
Non-target lesion	1 (1.6)	0	NA	
Heart failure	0	1 (1.6)	NA	

Values are presented as n(%). HR = hazard ratio; CI = confidence interval.

Table S3. Relationship between bleeding events and dyspnea.

Events	Severity	OR (95% CI)	*p* Value
Any BARC bleeding	Any dyspnea*<br>No dyspnea events	1.598 (1.008–2.533)<br>1	0.046
Any BARC bleeding	Mod-severe dyspnea*<br>No-mild dyspnea	1.611 (0.668–3.884)<br>1	0.289
Any BARC bleeding	≥ 3 Borg scale**<br>< 2 Borg scale	1.134 (0.876–1.468)<br>1	0.339
BARC bleeding (≥ type 2)	Mod-severe dyspnea*<br>No-mild dyspnea	0.530 (0.010–27.945)<br>1	0.754
BARC bleeding (≥ type 2)	≥ 3 Borg scale**<br>< 2 Borg scale	0.864 (0.260–2.891)<br>1	0.817

*as binary events, **as Borg scale category-ratio 10. BARC = bleeding academic research consortium; CI = confidence interval; OR = odds ratio.
